# Emergency airway management in the prone position: an observational mannequin-based simulation study

**DOI:** 10.1186/s41077-024-00285-4

**Published:** 2024-04-06

**Authors:** Wesley Rajaleelan, Eugene Tuyishime, Eric Plitman, Zoe Unger, Lakshmi Venkataraghavan, Michael Dinsmore

**Affiliations:** 1 Department of Anesthesia and Pain Management, The Ottawa Hospital, University of Ottawa, Ottawa, ON Canada; 2 Department of Anesthesia and Perioperative Medicine, Victoria Hospital, Western University, London, ON Canada; 3 Faculty of Medicine, University of Toronto, Toronto, ON Canada; 4 Department of Anesthesia and Pain Management, Toronto Western Hospital, University Health Network, University of Toronto, Toronto, ON Canada

**Keywords:** Prone extubation, Simulation, Mannequin

## Abstract

**Introduction:**

Accidental extubation during prone position can be a life-threatening emergency requiring rapid establishment of the airway. However, there is limited evidence of the best airway rescue method for this potentially catastrophic emergency. The aim of this study was to determine the most effective method to recover the airway in case of accidental extubation during prone positioning by comparing three techniques (supraglottic airway, video laryngoscopy, and fiber-optic bronchoscopy) in a simulated environment.

**Methods:**

Eleven anesthesiologists and 12 anesthesia fellows performed the simulated airway management using 3 different techniques on a mannequin positioned prone in head pins. Time required for definitive airway management and the success rates were measured.

**Results:**

The success rates of airway rescue were 100% with the supraglottic airway device (SAD), 69.6% with the video laryngoscope (CMAC), and 91.3% with the FOB. The mean (SD) time to insertion was 18.1 (4.8) s for the supraglottic airway, 78.3 (32.0) s for the CMAC, and 57.3 (24.6) s for the FOB. There were significant differences in the time required for definitive airway management between the SAD and FOB (*t* = 5.79, *p* < 0.001, 95% *CI* = 25.92–52.38), the SAD and CMAC (*t* = 8.90, *p* < 0.001, 95% *CI* = 46.93–73.40), and the FOB and CMAC (*t* = 3.11, *p* = 0.003, 95% *CI* = 7.78–34.25).

**Conclusion:**

The results of this simulation-based study suggest that the SAD I-gel is the best technique to manage accidental extubation during prone position by establishing a temporary airway with excellent success rate and shorter procedure time. When comparing techniques for securing a definitive airway, the FOB was more successful than the CMAC.

## Introduction

Accidental tracheal extubation during surgery is an acute emergency in the operating room and can be a life-threatening event if not followed by rapid re-establishment of the airway [1, 2]. This scenario becomes even more complicated when patients are positioned in the prone position and when undergoing spine or other neurosurgical procedures [2, 3]. Surgeries involving pathologies in the posterior fossa, suboccipital region, and posterior approaches to spine often require prone positioning. This is often logistically the most difficult positioning due to challenges associated with providing adequate oxygenation, ensuring adequate ventilation, and maintaining hemodynamic stability. Access to the patients’ airway is difficult and challenging especially when the head is fixed in flexion under Mayfield pins. Therefore, prone positioning for neurosurgical procedures might prove far more challenging compared to other procedures which warrant prone positioning.

Abrishami et al. in a systematic review found among 526 patients in 12 articles that the supraglottic airway device (SAD) was inserted successfully in the prone position in 87.5 to 100% of the patients. Moreover, ventilation was maintained successfully through the supraglottic device in 83.3 to 100% of all cases [1]. However, if a decision is made to establish a definitive airway, anesthesiologists have options to attempt reintubation in the prone position or treat this catastrophic event by turning the patient back to a supine position [3, 4]. There are currently very few studies in the literature examining emergency airway management in the prone position with a SAD or intubation to secure the airway [4–6].

Simulation-based training has shown to improve performance in clinical practice in anesthesia and surgery [7–9]. For example, laparoscopic skills in the operating room were better for residents with simulation video training in comparison to residents without simulation training [10]. Similarly, fiber-optic orotracheal intubation skills in the operating room were better for residents with simulation training in comparison to residents without simulation training [11, 12]. In addition, simulation has shown to be a good teaching modality for rare events [10]. The management of self-extubation in prone position is a rare event requiring advanced airway management skills which can be practiced and evaluated by simulation. However, anatomical limitations of mannequins to simulate real patients may lead to difficult translation of findings into clinical practice.

The aim of this study was to determine the success of airway management with a supraglottic airway device (SAD) I-gel, intubation with video laryngoscope (CMAC), and fiber-optic bronchoscope in a mannequin placed in the prone position under Mayfield pins intended to simulate a real-life scenario and to compare the time taken to manage airway successfully between these modalities.

## Material and methods

**Fig. 1:**
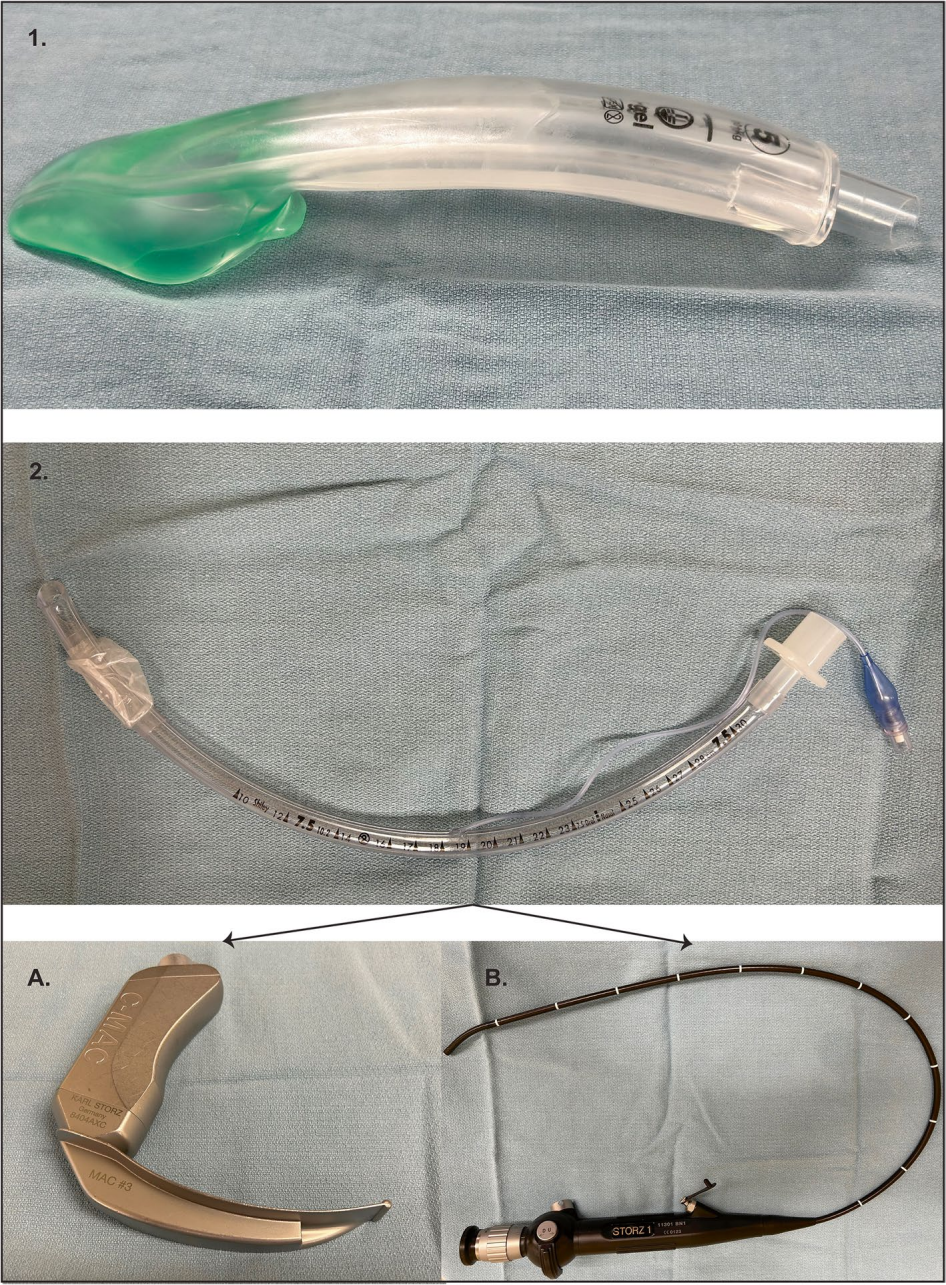
Airway devices used during the emergency airway management in the prone position simulation study. (1) I-gel supraglottic airway: it has a noninflatable cuff and a slightly curved tube to facilitate insertion. In addition, it has an independent gastric drainage tube and a bite block. (2) Endotracheal tube inserted using either the following: **A** C-MAC videolaryngoscopy: usually connected to a proprietary video display. **B** Fiber-optic airway: also connected to its own video display

**Fig. 2:**
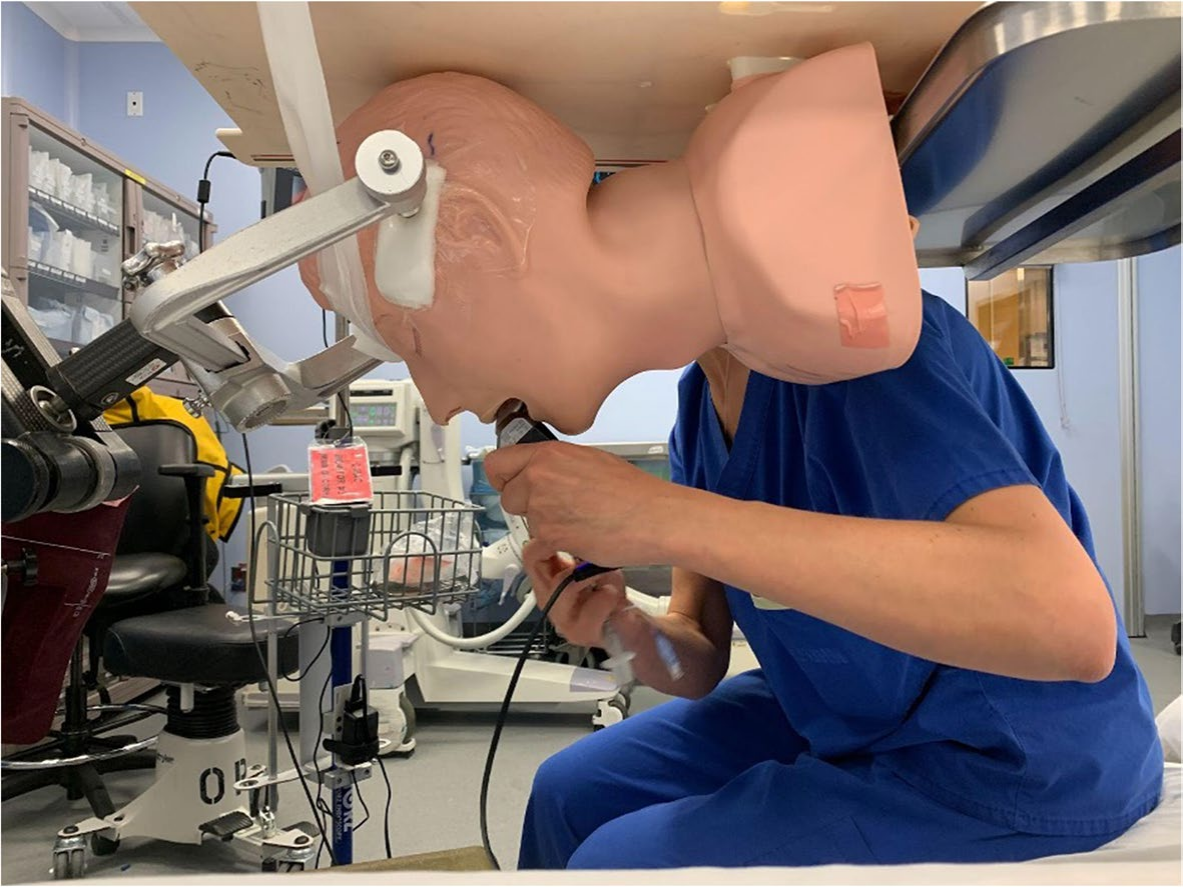
Mannequin fixed onto the OR table in a prone position with Mayfield pins mimicking position for posterior fossa surgery/cervical spine surgery

After institutional REB was reviewed and waived, the University Health Network (UHN) Quality Improvement Review Committee (QIRC) reviewed and approved the project (QI ID 21–0246) on Oct 4, 2021. After informed consent from the participants, we conducted an observational in situ simulation-based study comparing the airway rescue performances of three techniques: supraglottic airway (SAD), video laryngoscope (CMAC), and fiber-optic bronchoscope (FOB) (Fig. 1). Mayfield pins were placed on a mannequin (Laerdal airway Management Trainer, Norway) in the OR, which was fixed to the OR table, and the mannequin was turned into the prone position (Fig. 2).

Prior to the start of the scenario, participants were oriented by the study team to the simulation environment, equipment, and mannequin to ensure physical and psychological safety; then they were pre-briefed that there would be an accidental extubation in the prone position, and they would need to re-establish the airway. All the equipment was in the room and easily accessible. We only started the timing once the participant touched the equipment in order to exclude the time needed to get the equipment.

Then, the study team provided information about the scenario and requested participants to manage the airway using three techniques (supraglottic airway device I-gel (Intersurgical Ltd., UK), intubation with video laryngoscope (CMAC-Karl Storz, Germany), and fiber-optic bronchoscope (Karl Storz, Germany)) during accidental extubation of an adult patient undergoing a neurosurgical procedure in prone position.

A short debriefing at the end of the scenario of 5 min was performed for each participant to manage emotions and to ask for feedback.

Data was collected by two trained raters. The raters were trained on how to use the pre-designed data collection tool for 1 h by rating the same simulated procedures performed by the research team followed by time for questions and feedback. The data collection tool was developed based on previous studies done on fiber-optic intubation, laryngeal mask airway, and C-MAC video laryngoscope versus flexible fiber-optic scope in prone position, by Hung et al. (2008), Gupta et al. (2015), and Yumul et al. (2015) [3, 6, 13]. During data collection, one data collector focused on measuring the time variables using a timer displayed on a computer screen, while the second rater collected data on the remaining variables and contributed to the decision about the start time, end time, and failed attempt.

A total of 23 participants included staff anesthesiologists (11), and anesthesiology fellows (12) performed airway management, and the time required and the success rates were recorded. Staff anesthesiologists are physician anesthesiologists with independent license, while fellows work under supervision by staff anesthesiologists with an educational license while undergoing sub-specialty training. Success was confirmed by observation of bilateral inflation of mannequin lungs using an AMBU bag. Participants were given three attempts, and if any attempt was longer than 180 s, it was considered as a failed attempt. Anesthetic experience in years, prior experience of an inadvertent extubation during prone positioning, and technique of choice of securing the airway if faced with an inadvertent extubation were documented as part of the questionnaire.

### Sample size calculation

Currently, there is limited literature evaluating airway procedures in the prone position. Therefore, the sample size calculation was based on the comparison of the video laryngoscope (CMAC) to a flexible fiber-optic scope (FFS) in the supine position (Yumul et al. 2016) [13]. The time following initial insertion of laryngoscope blade to placement of the tracheal tube was 59 s and 35 s for FFS and CMAC, respectively, with a common standard deviation of 29 s. Therefore, to compare two independent means (two-sided test), using an alpha of 0.05 and a power of 0.8, the sample size for this study was 23 participants.

In addition, recent study comparing McGrath and SAD insertion in the prone position calculated a sample size of 19 participants would be needed based on a 15-s difference between devices and a SD of 14 s [14]. Statistical analysis was carried out using SPSS version 28.0 (IBM, USA). Data were presented as mean ± SD, as appropriate. Notably, data from failed trials in the present study were not included in mean time required for definitive airway management calculations. Linear mixed-effects models were used to test the difference in mean time required for definitive airway management (mean of non-failed trials within modality) between SAD, CMAC, and FOB, with modality as a fixed effect and subject as a random effect. The influence of participants’ position (staff or fellow), prior experience of handling an inadvertent prone extubation (yes or no), and number of years of experience were tested through their inclusion in the aforementioned model. A significance level of *p* < 0.05 was employed.

## Results

Twenty-three participants (11 (47.8%) staff anesthesiologists and 12 (52.1%) anesthesia fellows) were recruited for this study. Five (23.8%) participants (2 staff and 3 fellows) had a prior experience of handling an inadvertent prone extubation in their career. The mean experience of the participants in anesthesiology as a specialty was 12.0 (5.8) years.

The mean (SD) time taken to secure the airway was 18.1 (4.8) s for the SAD, 78.3 (32.0) s for the CMAC, and 57.3 (24.6) s for the FOB.

The mean (SD) time taken to secure the airway using the SAD was 18.6 (5.0) s amongst the fellows and 17.6 (4.7) s amongst the staff. The mean time taken to secure the airway using the FOB was 65.8 (24.7) s amongst the fellows and 48.0 (22.0) s amongst the staff. The mean time taken to secure the airway using the CMAC was 68.7 (32.6) s amongst the fellows and 88.8 (29.0) s amongst the staff (Table 1).

**Table 1:** Mean average time taken to establishing a definitive airway

Airway	Position	Mean average time to insertion in s (SD)
SAD-i GEL	Fellow	18.6 (5.0)
	Staff	17.6 (4.7)
CMAC	Fellow	68.7 (32.6)
	Staff	88.8 (29.0)
FOB	Fellow	65.8 (24.7)
	Staff	48.0 (22.0)

*SAD* supraglottic airway device, *CMAC* videolaryngoscope, *FOB* fiber-optic bronchoscope

The mean (SD) time taken to establish a successful airway using the SAD was 18.2 (3.8) s in participants with no prior experience of handling an inadvertent prone extubation and 17.9 (8.3) s in participants with prior experience of handling an inadvertent prone extubation. The mean time taken to secure the airway using the FOB was 60.5 (26.7) s in participants with no prior experience of handling an inadvertent prone extubation and 47.4 (15.6) s in participants with prior experience of handling an inadvertent prone extubation. The mean time taken to secure the airway using the CMAC was 78.0 (36.6) s in participants with no prior experience of handling an inadvertent prone extubation and 78.2 (21.1) s in participants with prior experience of handling an inadvertent prone extubation (Table 2).

**Table 2:** Mean average time to establishing a definitive airway compared to previous experience of the operator in handling an inadvertent prone extubation

Airway	Previous experience of inadvertent prone extubation (number of participants)	Mean average time to insertion in sec (SD)
SAD	No (16)	18.2 (3.8)
	Yes (5)	17.9 (8.3)
CMAC	No (16)	78.0 (36.6)
	Yes (5)	78.2 (21.1)
FOB	No (16)	60.5 (26.7)
	Yes (5)	47.4 (15.6)

*SAD* supraglottic airway device, *CMAC* video laryngoscope, *FOB* fiber-optic bronchoscope

**Fig. 3:**
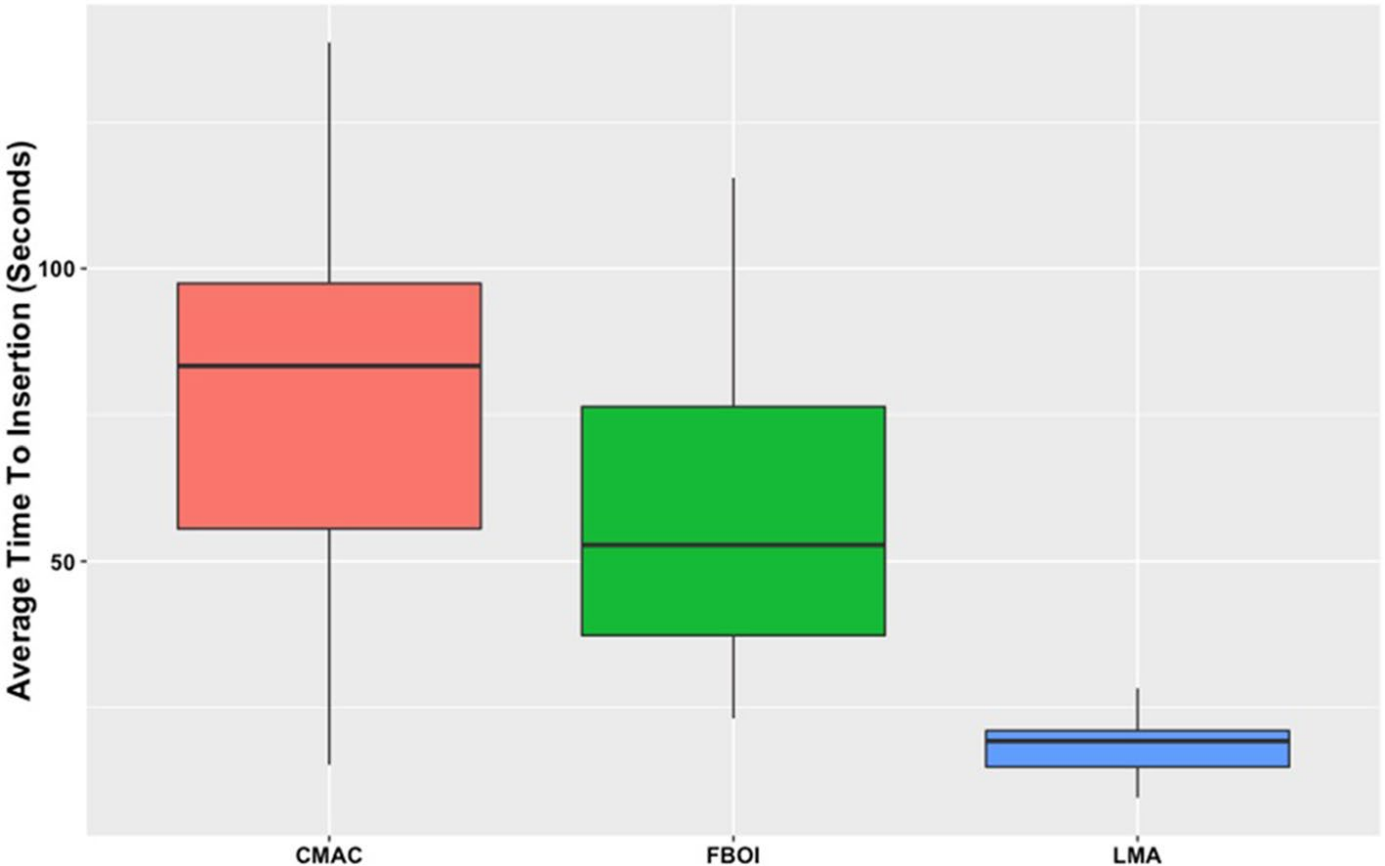
Differences in time taken to intubate using I-gel laryngeal mask airway (LMA). Videolaryngoscope (CMAC) and fiber-optic bronchoscope

There were significant differences in the time required for successful airway management between the SAD and FOB (*t* = 5.79, *p* < 0.001, 95% *CI* = 25.92–52.38), the SAD and CMAC (*t* = 8.90, *p* < 0.001, 95% *CI* = 46.93–73.40), and the FOB and CMAC (*t* = 3.11, *p* = 0.003, 95% *CI* = 7.78–34.25) (Fig. 3). The inclusion of participants’ position, prior experience of handling an inadvertent prone extubation, or number of years of experience as covariates in the model did not affect results.

All the participants were successful in establishing a SAD as a temporary salvaging device. Seven participants failed at least one trial to establish a definitive ET using the CMAC, including five fellows and two staff, and three participants with prior experience of handling an inadvertent prone extubation. Two participants failed at least one trial to establish a definitive ET using the FOB, including one fellow and one staff, and no participants with prior experience of handling an inadvertent prone extubation (Table 3).

**Table 3:** Failed attempts to establish a definitive airway

Airway	Successful attempts	Failed attempts
SAD	23	0
CMAC	16	7
FOB	21	2

*SAD* supraglottic airway device, *CMAC* video laryngoscope, *FOB* fiber-optic bronchoscope

In the post-study questionnaire on the preference of the airway rescue device if faced with an acute emergency of prone inadvertent extubation, 5 participants chose the CMAC, and 18 chose the FOB to aid in establishing a definitive airway.

## Discussion

In this simulation study, a SAD was successfully inserted by all the participants, and the time for SAD insertion was shorter than intubation with both a CMAC and FOB. There were fewer failures with SAD in comparison to intubation with CMAC and FOB. Regarding intubation, FOB had more success than video laryngoscopy.

Accidental prone extubation can be an acute emergency. A SAD can be used as a temporary airway, and the CMAC and FOB can be used to establish a definitive airway with an ET. Our study results confirm that SAD is the fastest rescue technique in the case of unexpected extubation during prone positioning albeit being only a temporary method. We chose the I-gel as the SAD based on the study by Gupta et al. where they compared the most appropriate SAD for initial airway management in a prone position during accidental extubation [6]. They compared the I-gel, ProSeal laryngeal mask airway (LMA), and the classic LMA [6]. They found that the time taken for I-gel insertion was significantly lesser (12.89 ± 3.94 s) as compared to classic LMA (17.07 ± 3.5 s) and ProSeal LMA (25 + 4.78 s) [6]. In addition, the other airway equipment like the VL and the FOB should be easily accessible as the SAD is not a definitive airway in a patient who is positioned prone [15–17].

It may be reasonable with appropriate training in intubation with CMAC in nonstandard positions, to consider VL as a first airway rescue option with the advantage to establish a definitive airway without the risk of SAD malfunction. Apneic oxygenation techniques should be considered to allow more time to safely secure the airway [17].

We also reported that the fellows were slower using a FOB compared to the staff. This is likely a result of performing less FOBs compared to staff anesthesiologists.

At the conclusion of this study, when enquired about the preference of the airway the participants would choose if faced with an acute emergency of prone inadvertent extubation, 5 participants chose the CMAC, and 18 chose the FOB to aid in establishing a definitive airway. This illustrates that participants were comfortable using the FOB as the primary rescue method of securing the airway with an ET. Even if previous experience was not assessed, all three techniques are part of routine practice at our institution, and we have included full trained anesthesiologists with competency in all three techniques; therefore, we believe that previous experience in using these techniques was not different among participants and did not influence participants’ performance or choice of the preferred technique as shown by their choice of the FOB technique which is less commonly used and more complex to teach.

With appropriate technique, the patient can be safely turned to spine position; however, there is increased risk of contamination of the surgical field, and this should be done as a last option if the airway rescue is found challenging. Our study is validating previous algorithms for management of self-extubation in prone position suggesting using SAD as first option followed by the video laryngoscope or fiber optic such as the one developed by Gaszynski in 2020 [18]. SAD provides an added advantage of being readily available in most operating rooms and easy to use; this has potential to minimize time without adequate oxygenation and allow enough time to prepare resources needed to establish a definitive airway based on different factors such as patient characteristics (i.e., obesity, respiratory or cardiac diseases) and type and progress of the procedure (i.e., multi-level spine surgery at the beginning, single-level spine surgery towards the end of the operation). Gaszynski proposed a summarized algorithm for the management of unexpected extubation during prone position including calling for help, preparing airway equipment and changing position to supine, and rescuing airway with either SAD or FOB or channelled VL [18].

This algorithm provides systematic approach and guidance while allowing flexibility for clinical judgement according to the patient status, availability of resources, and level of training in airway management [18].

The major strength of this study is using simulation to study an important and rare clinical event and contributing to the existing evidence about using simulation to inform clinical performance [7–9]. We have chosen simulation method for this project because the event of self-extubation in prone position is rare, and the set of skills needed is narrow allowing easy teaching and assessment [9]. Other rare events have been taught with simulation such as maternal and neonatal resuscitation and management of cardiac arrest [19, 20]. For example, Lipman and colleagues in 2013 used the simulated case of uterine rupture to determine the time required to move the patient from the labor room to the OR (9 min and 27 s) and identified essential intervals (decision to move, exiting the labor room, transit from labor room to OR, time to intubation, and time from intubation to incision) to determine system-related weaknesses [19]. In addition, Hards and colleagues in 2012 used a simulated scenario of maternal cardiac arrest to assess performance of residents after didactic versus e-learning training showing overall improvement in time to perimortem cesarean delivery (PMCD) (from 4.5 to 3.5 s), better technical scores (i.e., PMCD, cardiopulmonary resuscitation with pregnancy modifications), and better nontechnical skills (i.e., task management, decision-making, situation awareness, teamwork) scores [20]. These two examples used simulation to assess performance while managing rare events as done in our study to determine the best airway management approach in managing the case of self-extubation in prone position. However, in our study, we did not test team performance rather individual performance; this can negatively impact performance while translating our findings into clinical practice where team management is necessary. But, as our participants were fully trained anesthesiologists with prior team training, the effect may be minimal.

There are some limitations to consider while interpreting the results of this study. The sample size was small, and mannequins have different limitations while simulating a real-life scenario. There are anatomical limitations associated with the use of mannequins (i.e., inability to simulate secretions, different patient characteristics like obesity or difficult airway) which may lead to higher failure rate in real clinical situations despite high rates of success with all three equipment (SAD, CMAC, and fiber optic) in our study [16, 17]. In addition, having all airway equipment available to participants before airway management is unlikely to happen in clinical practice due to resources constraints; this will impact the translation of the findings of our study into clinical practice based on available resources at each center. In our tertiary center, SAD and video laryngoscopes are readily available; however, the fiber-optic device may take more time to bring to the operating room and to set it up. Furthermore, our detailed pre-brief about the expected event could have decrease the stress and improved performance. This has an impact on translation of our findings into practice because the self-extubation in prone position in real life would lead to stress and emotions which would decrease performance as explained by LeBlanc in 2022 [21]. Finally, as our main objective was to compare the three techniques of airway management, we did not assess participants’ interpersonal skills such as situation awareness, decision-making, teamwork, and task management, which would have a negative impact on clinical performance in case of breakdown of any of these skills.

## Conclusion

The results of this simulation-based study suggest that the SAD I-gel is the best technique to manage accidental extubation during prone position by establishing a temporary airway with excellent success rate and shorter procedure time. When comparing techniques for securing a definitive airway, the FOB was more successful than the CMAC.

This study has potential to inform the design of the simulation curriculum for training the perioperative team (i.e., nurses, surgeons, anesthesiologists, residents, and fellows) involved in managing patients undergoing surgery in prone position. Further studies should investigate the best strategy to translate the findings of this simulation-based study into clinical practice.

## Glossary

LMA: Laryngeal mask airway
SAD: Supraglottic airway device
CMAC: Video laryngoscope
ETT: Endotracheal tube
OR: Operating room
FOB: Fiber-optic bronchoscope
NTS: Nontechnical skills
